# Supplementary material for: RGS4 RNA Secondary Structure Mediates Staufen2 RNP Assembly in Neurons
Source: Int J Mol Sci. 2021 Dec 1;22(23):13021. doi: 10.3390/ijms222313021 (PMC8657808; doi:10.3390/ijms222313021)
Supplement: Supplementary file 1 [file ijms-22-13021-s001.zip › ijms-1447368-supplementary.pdf]

International Journal of *Molecular Sciences*

MDPI

Supplementary Materials: The following are available online at www.mdpi.com/arti-cle/10.3390/ijms222313021/s1.

Supplementary Figure 1

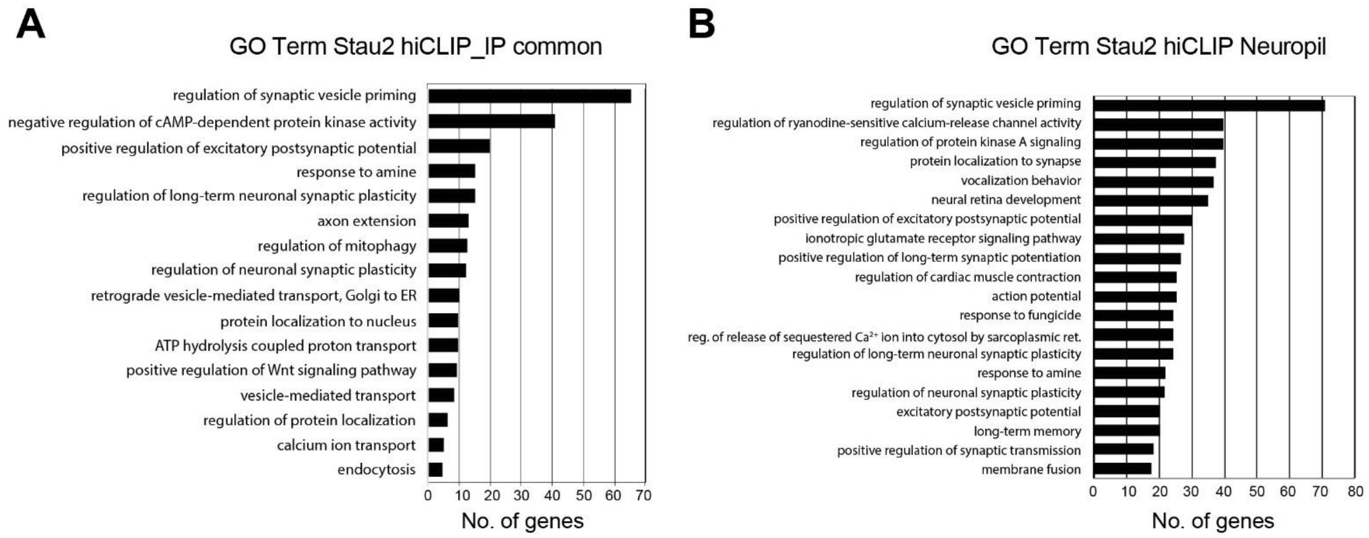

Figure S1. Stau2-RNA duplexes are enriched in dendritically localized transcripts with extended 3′-UTRs (corresponds to Fig. 1). (A) GO term enrichment analysis of shared Stau2 mRNAs from previously published Stau2 immunoprecipitation [1] and the new Stau2 hiCLIP data. (B) GO term enrichment analysis of regulated Stau2 mRNAs that are enriched in the neuropil [2] (P35 brain) indicated in Fig. 1 D..

Supplementary Figure 2

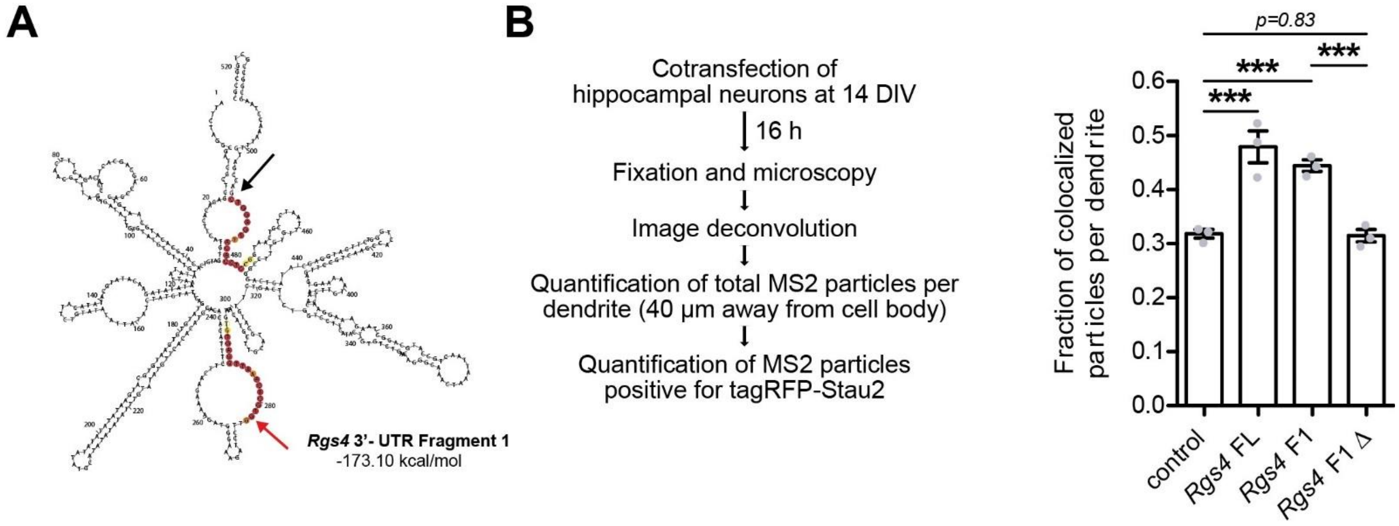

Figure S2. *Rgs4* RNA duplex is sufficient to drive Stau2-RNP assembly (corresponds to Fig. 2). (**A**) Predicted *in silico* folding of *Rgs4 fragment 1*. The two arms of the hairpin identified by hiCLIP are shown in red. Arrows indicate the starting 5′- part (red) and end 3′- part (black) positions of the structure. (**B**) Experimental outline for the co-localization experiment (left) and quantification (right) of co-localization between tagRFP-Stau2 and MS2 particles relative to total number of MS2

particles in entire dendrites per experiment, starting 40 µm away from the cell body. Mean ± SEM from three independent biological replicates (shown as individual dots). Paired students t-test. Asterisks represent p-values ***p < 0.001.

Supplementary Figure 3

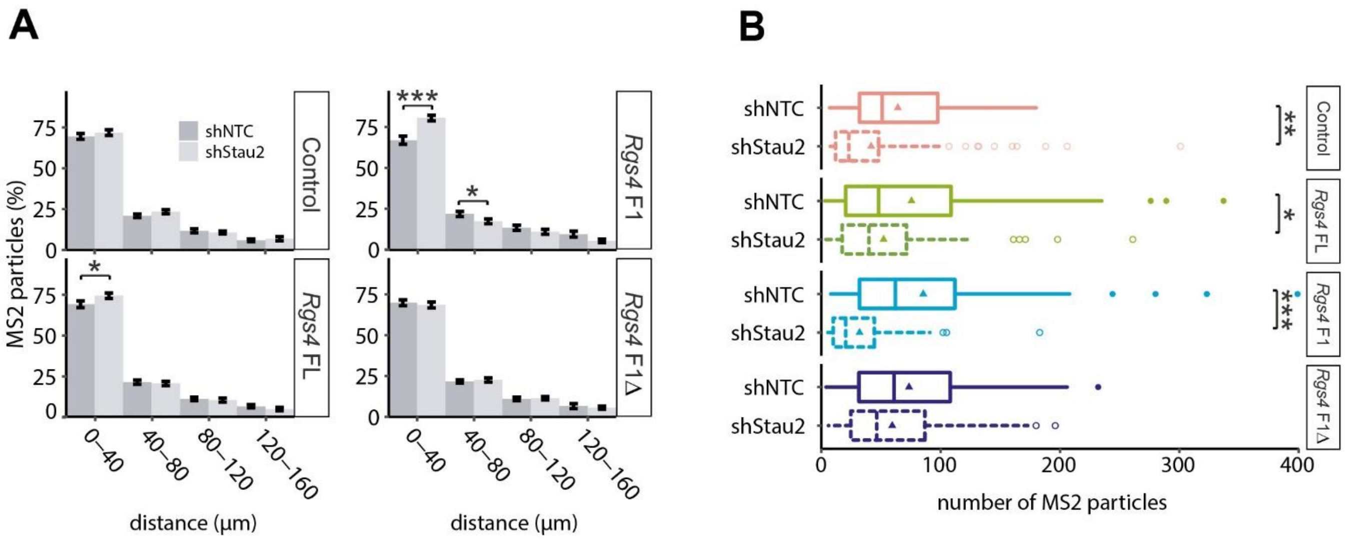

Figure S3. Stau2 mediates RNA structure dependent dendritic localization (corresponds to Fig. 3). (**A**) Histogram displaying percentage of MS2 particles per dendrite in 40 µm bins along dendrites of hippocampal neurons transfected with tdMCP-GFP and 128xMS2. Panel corresponds to Fig. 3B. (**B**) Boxplot of the average number of MS2 particles per dendrite. Unpaired student's t-test. Data are obtained from 4 independent biological replicates with at least 15 dendrites per replicate. Asterisks represent p-values (*p < 0.05, **p < 0.01, ***p < 0.001). FL, full length; F1 fragment 1; F1Δ fragment 1 with a 20 nt deletion; shNTC, control; shStau2, Stau2 KD conditions.

Supplementary Figure 4

A

Mapt

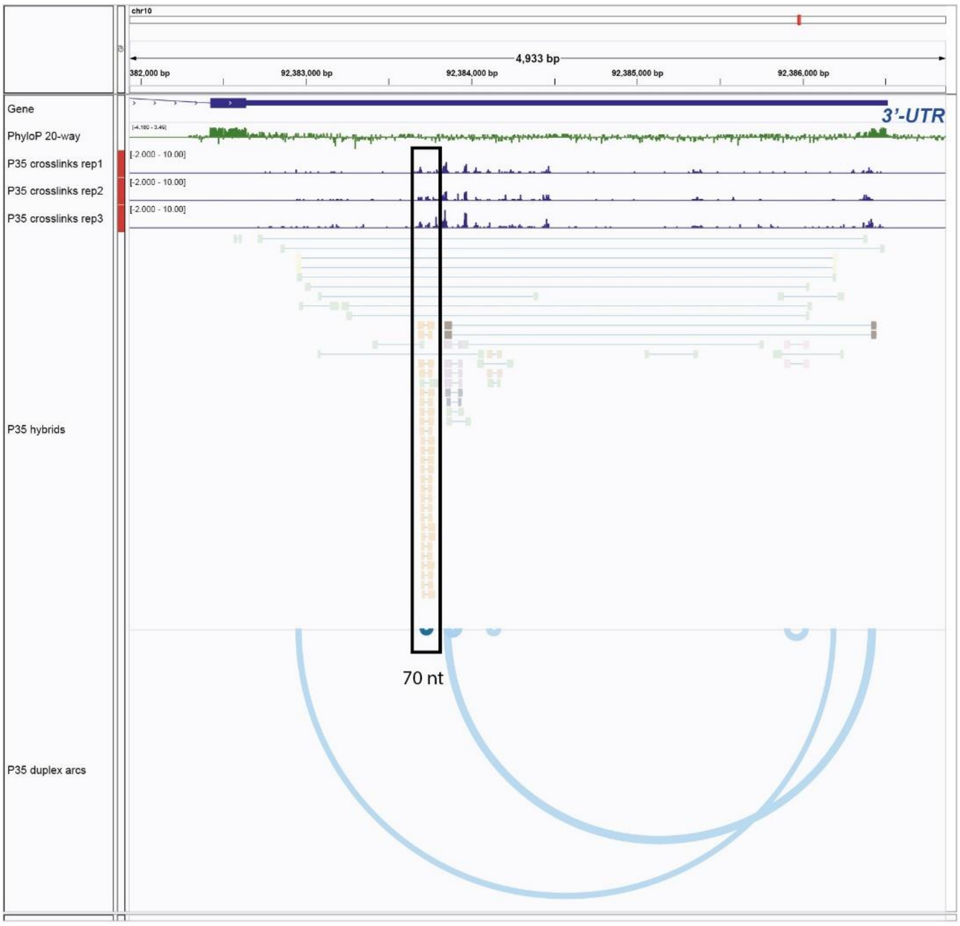

B

RhoA

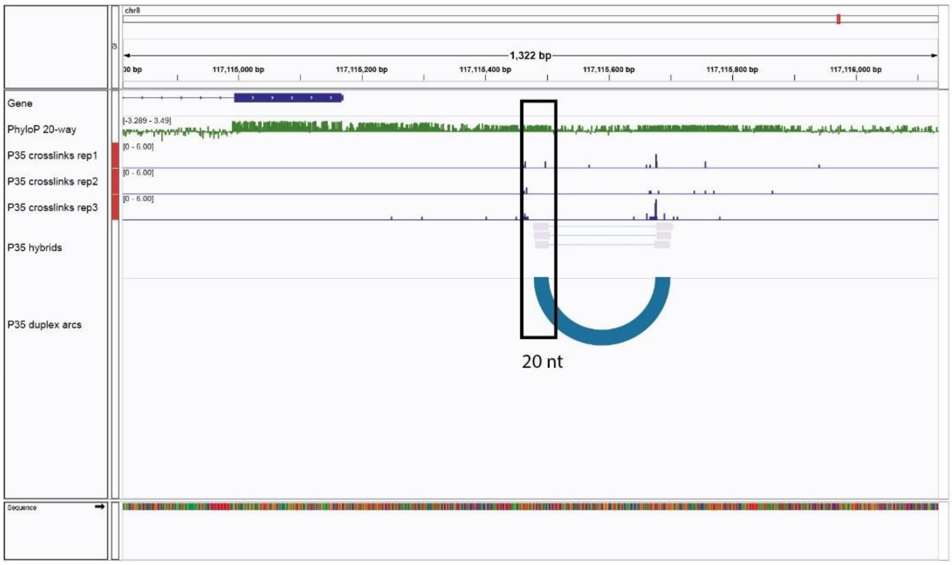

Supplementary Figure 4ff

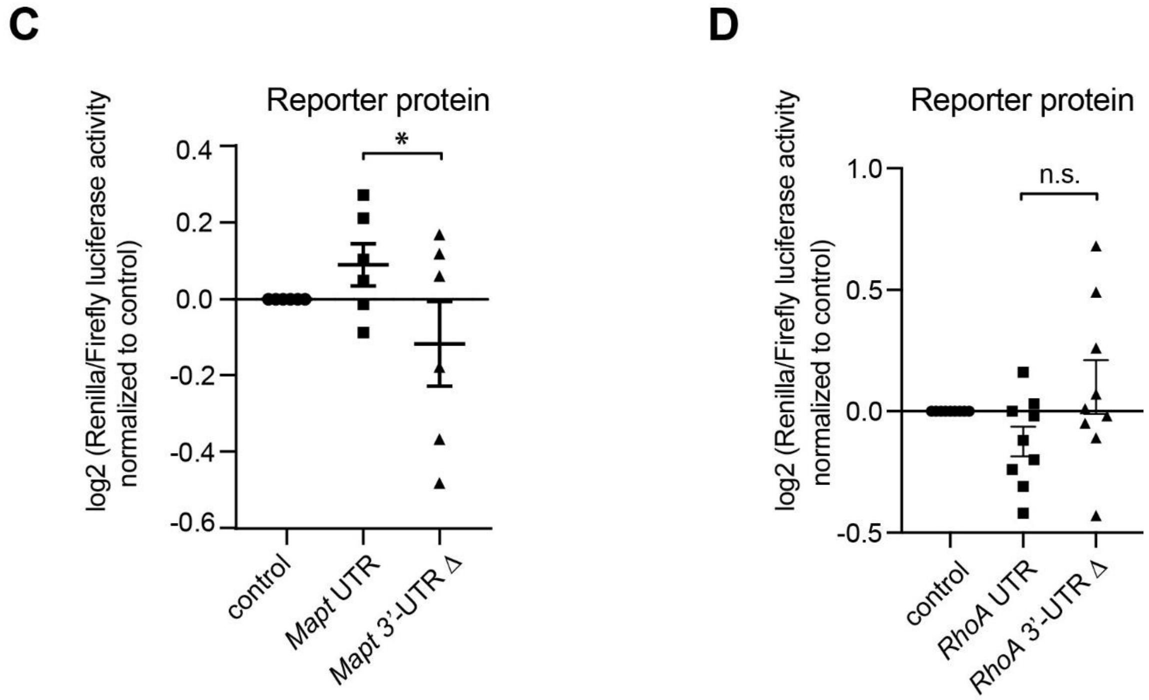

Figure S4. RNA duplexes alter Stau2-dependent reporter expression in neurons (corresponds to Fig. 4). (**A** and **B**) Coverage of Stau2-hiCLIP (P35 hybrids) and iCLIP (P35 crosslinks) sites in *Mapt* and *RhoA* 3′-UTR (blue) is depicted together with the PhyloP (100x vertebrates) sequence conservation (green) and the duplex linking arc plot. One arm of the Stau2-duplex is deleted in each full length 3′-UTR (Δ). Location (vertical black box) and size are highlighted. (**C** and **D**) Luciferase (RRL/FFL) activity in primary rat cortical neurons at 7+1 DIV transfected with the indicated control (no UTR), *Mapt* 3′-UTR (**C**) or *RhoA* 3′-UTR (**D**) or Stau2-duplex deletion (Δ) constructs. All data is normalized to control. Kolmogorov-Smirnov test combined with paired student's t-test. Error bars represent SEM from at least 3 independent biological replicates. Asterisks represent p-values *p < 0.05; n.s., non significant.

References

1. Heraud-Farlow, J.E., et al., *Staufen2 regulates neuronal target RNAs.* Cell Rep, 2013. **5**(6): p. 1511-8.
2. Cajigas, I.J., et al., *The local transcriptome in the synaptic neuropil revealed by deep sequencing and high-resolution imaging*. Neuron, 2012. **74**(3): p. 453-66.
